# Glycosaminoglycans: From Vascular Physiology to Tissue Engineering Applications

**DOI:** 10.3389/fchem.2021.680836

**Published:** 2021-05-18

**Authors:** Antonio Junior Lepedda, Gabriele Nieddu, Marilena Formato, Matthew Brandon Baker, Julia Fernández-Pérez, Lorenzo Moroni

**Affiliations:** ^1^ Department of Biomedical Sciences, University of Sassari, Sassari, Italy; ^2^ Complex Tissue Regeneration Department, MERLN Institute for Technology Inspired Regenerative Medicine, Maastricht, Netherlands

**Keywords:** glycosaminoglycans, scaffolds, tissue engineering, vascular regeneration, vascular disease

## Abstract

Cardiovascular diseases represent the number one cause of death globally, with atherosclerosis a major contributor. Despite the clinical need for functional arterial substitutes, success has been limited to arterial replacements of large-caliber vessels (diameter > 6 mm), leaving the bulk of demand unmet. In this respect, one of the most challenging goals in tissue engineering is to design a “bioactive” resorbable scaffold, analogous to the natural extracellular matrix (ECM), able to guide the process of vascular tissue regeneration. Besides adequate mechanical properties to sustain the hemodynamic flow forces, scaffold’s properties should include biocompatibility, controlled biodegradability with non-toxic products, low inflammatory/thrombotic potential, porosity, and a specific combination of molecular signals allowing vascular cells to attach, proliferate and synthesize their own ECM. Different fabrication methods, such as phase separation, self-assembly and electrospinning are currently used to obtain nanofibrous scaffolds with a well-organized architecture and mechanical properties suitable for vascular tissue regeneration. However, several studies have shown that naked scaffolds, although fabricated with biocompatible polymers, represent a poor substrate to be populated by vascular cells. In this respect, surface functionalization with bioactive natural molecules, such as collagen, elastin, fibrinogen, silk fibroin, alginate, chitosan, dextran, glycosaminoglycans (GAGs), and growth factors has proven to be effective. GAGs are complex anionic unbranched heteropolysaccharides that represent major structural and functional ECM components of connective tissues. GAGs are very heterogeneous in terms of type of repeating disaccharide unit, relative molecular mass, charge density, degree and pattern of sulfation, degree of epimerization and physicochemical properties. These molecules participate in a number of vascular events such as the regulation of vascular permeability, lipid metabolism, hemostasis, and thrombosis, but also interact with vascular cells, growth factors, and cytokines to modulate cell adhesion, migration, and proliferation. The primary goal of this review is to perform a critical analysis of the last twenty-years of literature in which GAGs have been used as molecular cues, able to guide the processes leading to correct endothelialization and neo-artery formation, as well as to provide readers with an overall picture of their potential as functional molecules for small-diameter vascular regeneration.

## Introduction

Cardiovascular diseases are a group of disorders affecting heart and blood vessels that represent a significant global health problem, being the leading cause of morbidity and mortality in the world. In 2019 acute clinical events, such as heart attack and stroke, mainly caused by the atherosclerosis of coronaries, carotid, and cerebral arteries, have been the cause of 16.2% and 11.6% of the global deaths, respectively (Data from the Global Burden of disease, https://vizhub.healthdata.org/gbd-compare/). Atherosclerosis is a chronic systemic inflammatory condition with a very complex etiology in which both genetic (e.g. hypertension, diabetes, obesity, high blood cholesterol and other lipids) and environmental (e.g. lifestyles including unhealthy diet, physical inactivity, smoking, alcohol abuse) risk factors play a role (Benjamin et al., 2017). Atherosclerosis is characterized by the accumulation of lipids and fibrous elements into the subendothelial space of arteries leading to plaque formation, which, ultimately, could evolve into an acute clinical event due to plaque rupture and thrombosis (Libby et al., 2019). There are many evidences demonstrating that high levels of plasma cholesterol-rich lipoproteins (LDLs) have a causal role in the early events leading to lesion formation (atherogenesis) (Ference et al., 2017). In this respect, it is known that some vascular extracellular matrix (ECM) components mediate their entrapment into the subendothelial space, thus leading to lipids accumulation within the arterial intima.

A major commitment of vascular tissue engineering is to design biomimetic scaffolds that combine the mechanical properties of natural blood vessels with biocompatibility, controlled biodegradability, and a specific combination of molecular signals allowing for vascular regeneration. Despite the clinical need for functional arterial substitutes, success has been limited to arterial replacement of large- and medium-caliber vessels with Dacron (polyethylene terephthalate) and Goretex (expanded polytetrafluoroethylene) synthetic grafts, leaving the bulk of demand unmet. In fact, for the replacement of small-diameter vessels (< 6 mm), such synthetic grafts are often rejected in a few months due to restenosis; hence, autologous veins (e.g. the great saphenous vein) or arteries (e.g. the internal mammary artery, and the radial artery) remain the best choice, despite their limited availability. To overcome these limitations, in the last decades, different tissue engineering approaches have been applied to develop vascular substitutes using natural, synthetic or hybrid materials, some of them reaching the preclinical/clinical stages (Liu et al., 2018).

Several attempts were performed to create completely biological living substitutes, such as collagen- or fibrin-based scaffolds in combination with vascular cells, sometimes with promising results (Peck et al., 2012). The approach of tissue engineering by self-assembly (TESA), allowing for the construction of an engineered blood vessel starting from autologous fibroblasts, obtained from skin biopsy, has been proven to be useful as arterial bypass grafts in long-term animal models (L'Heureux et al., 2006) as well as arteriovenous shunt in hemodialysis patients (L'Heureux et al., 2007). However, besides the high costs of fabrication, the long manufacturing time (i.e. few months) represents a limitation in patients who need rapid intervention. Interesting results were recently obtained by using acellular tissue engineered vessels (A-TEV) (Koobatian et al., 2016; Smith et al., 2019; Smith et al., 2020). This technology relies on decellularized natural matrices functionalized with bioactive molecules, thus providing host cells with a physiological environment. Issues remain about the total removal of xenogenic material and decellularizing agents that could elicit an immune response.

Several synthetic vascular grafts have been developed with the aim to guide vascular cells to attach, proliferate, and synthesize their own ECM, in which the inflammatory/thrombotic nature of the scaffolds could be overcome by coating them with bioactive natural molecules (Pashneh-Tala et al., 2016). Fabrication methods, such as phase separation, self-assembly and electrospinning, have been used to obtain biocompatible nanofibrous scaffolds with a well-organized architecture and mechanical properties suitable for vascular tissue regeneration (Yao et al., 2020). However, several evidences have shown that naked scaffolds for small-diameter blood vessel replacement suffer from low patency rates, are pro-thrombotic, susceptible to infection and do not have growth potential for the pediatric population (Song et al., 2018). Furthermore, they often represent a poor substrate to be populated by vascular cells (Awad et al., 2018). To overcome these issues, variously functionalized bioresorbable scaffolds have been developed as temporary guides for neo-artery formation. In this respect, surface functionalization with drugs or bioactive natural molecules, including ECM components such as collagen, elastin, or glycosaminoglycans (GAGs), and growth factors, has been proven to be effective in reducing thrombotic events, enhancing endothelialization, and further promoting cell proliferation.

Here, the composition of normal blood vessel and some pathophysiological roles of vascular proteoglycans (PGs) and GAGs have been discussed. Besides, the potential of GAGs as functional molecules for vascular tissue regeneration is the main topic of this review. In this respect, we performed an in-depth literature search, using MEDLINE (PubMed), with the aim of providing readers with the main findings of both *in vitro* and *in vivo* studies on the different small-diameter vascular devices, functionalized with GAGs, developed in the last 20 years.

## Significance of Proteoglycans and Glycosaminoglycans in Vascular Biology

### Blood Vessel Wall Structure

Blood vessels share some common features as they all consist of three concentric layers called tunica Intima, facing the vessel lumen, tunica Media and tunica Adventitia. The Intima is composed by a continuous monolayer of endothelial cells anchored to the basement membrane, which is involved in critical events such as blood coagulation, exchange of oxygen and nutrients, vascular tone regulation through mechanosensing (induction of endothelial nitric oxide synthesis by shear stress), inflammation, and immune response (Kruger-Genge et al., 2019). Most of the Intima’s functions are modulated by the endothelial glycocalyx, a dynamic and complex gel-like network on the luminal surface of endothelial cells, consisting of PGs (syndecans, glypicans), glycoproteins, GAGs (hyaluronan, heparan sulfate and chondroitin sulfate) and soluble proteins from both plasma and endothelium. The thickness (approximately 0.5–5.0 μm) and structure of the glycocalyx vary in relation to the vascular bed, blood flow rate, pathophysiological conditions, and they result from a dynamic balance between constant biosynthesis of new glycans and shear-dependent alterations, with a considerable rate of turnover of its components (Machin et al., 2019). Derangement of the endothelial glycocalyx structure plays major roles in several pathological conditions, including cardiovascular disease (Machin et al., 2019), diabetes (Dogne et al., 2018), kidney disease (Jourde-Chiche et al., 2019), sepsis (Uchimido et al., 2019), and trauma (Tuma et al., 2016). Underneath the tunica Intima there is the Media, a thick contractile multilayer of smooth muscle cells that provide support to the vessel as well as participate in regulating both blood flow and pressure. The Adventitia, with its collagenous ECM, is primarily responsible for the tensile strength of blood vessels. The three layers are separated from each other by an internal elastic lamina (between Intima and Media) and an external elastic lamina (between Media and Adventitia), consisting of fibers that provide elasticity to the vessel wall. In this scenario, the ECM in which vascular cells are immersed, mainly composed of collagens, elastin, fibronectin, laminins, glycoproteins, and PGs, not only provides the vascular wall with its mechanical properties, but also plays crucial roles in vascular cells homeostasis and pathogenesis. Therefore, the vascular wall is a very complex multilayer structure, in which different resident and circulating cell types interact with each other and with the ECM. Designing a substitute able to mimic the mechanical properties as well as the physiological functions of the vascular wall represents a challenge for tissue engineering.

### Proteoglycans and Glycosaminoglycans of the Vascular Wall

PGs are hydrophilic molecules, which provide the vasculature with viscoelasticity and turgor. These molecules participate in a number of vascular events such as regulation of vascular permeability, lipid metabolism, hemostasis, and thrombosis, as well as interact with vascular cells, growth factors, and cytokines, in order to modify vascular cell adhesion, migration, and proliferation. Some PGs exert specific functions in their soluble forms following cleavage by proteolytic enzymes (Lepedda et al., 2021). The amount of PGs in the artery wall is physiologically low, but increases deeply during the early phases of atherosclerotic lesion formation. Many studies have shown that PGs are involved in retention of cholesterol-rich lipoproteins and other serum components, ECM metabolism, and crosstalk with inflammatory cells that extravasate to the subendothelial space. Readers are referred to the excellent review by Wight TN (Wight, 2018) for a more detailed discussion on this topic.

**FIGURE 1:**
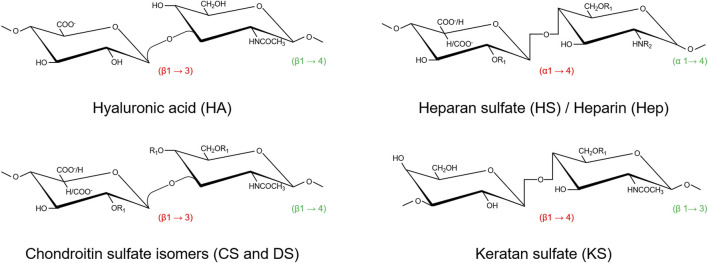
Structures of the repeating disaccharide units representative of the six GAGs classes [modified from (Vynios et al., 2002)]. Both DS and HS/Hep are co-polymers of two types of disaccharide repeats where glucuronate is variably substituted by its carbon-5 epimer iduronate. Hep has both a higher degree of sulfation and epimerization than HS. Except for HA, sulfation may occur in several positions, thus giving these glycans a characteristic high negative charge. Saccharides are reported as chair conformations. Configurations of the O-glycosidic bonds are reported in red (within disaccharide units) and in green (between adjacent disaccharide units). R_1_ = SO_3_^−^; R_2_ = COCH_3_/SO_3_^−^.

PGs have common structural features, as they all consist of a core protein covalently linked to one or more GAG chains, primarily responsible for their biological properties (Raman et al., 2005). GAGs are a family of anionic heteropolysaccharides that differ in terms of type of repeating disaccharide units, chains length, charge density, degree of sulfation, and hexuronic acid epimerization, found in connective tissues as well as in biological fluids such as plasma and urine (Vynios et al., 2002). The repeating disaccharide units are composed by a N-acetylated hexosamine (N-acetylgalactosamine or N-acetylglucosamine) and a hexuronic acid (glucuronic acid or its carbon-5 epimer iduronic acid) or galactose (in keratan sulfate). GAGs are key structural and functional components of the ECM of connective tissues, playing numerous biological roles, including embryonic development, ECM assembly and regulation of cell signaling in various physiological and pathological conditions. Indeed, their numerous functions are the result of their large structural heterogeneity, 202 unique disaccharide units have been identified in mammals (Clerc et al., 2019), responsible for the binding of a plethora of proteins including cytokines and chemokines, enzymes and enzyme inhibitors, ECM proteins, and membrane receptors (Kjellen and Lindahl, 2018). A comprehensive GAG-interactome composed of 827 proteins has been recently published (Vallet et al., 2021). Six main classes of GAGs have been described so far: hyaluronan or hyaluronic acid (HA), chondroitin sulfate (CS) and dermatan sulfate (DS), keratan sulfate (KS), heparan sulfate (HS), and heparin (Hep) (Figure 1); all GAG classes were found in normal and diseased arteries (Wight, 1980).

**FIGURE 2:**
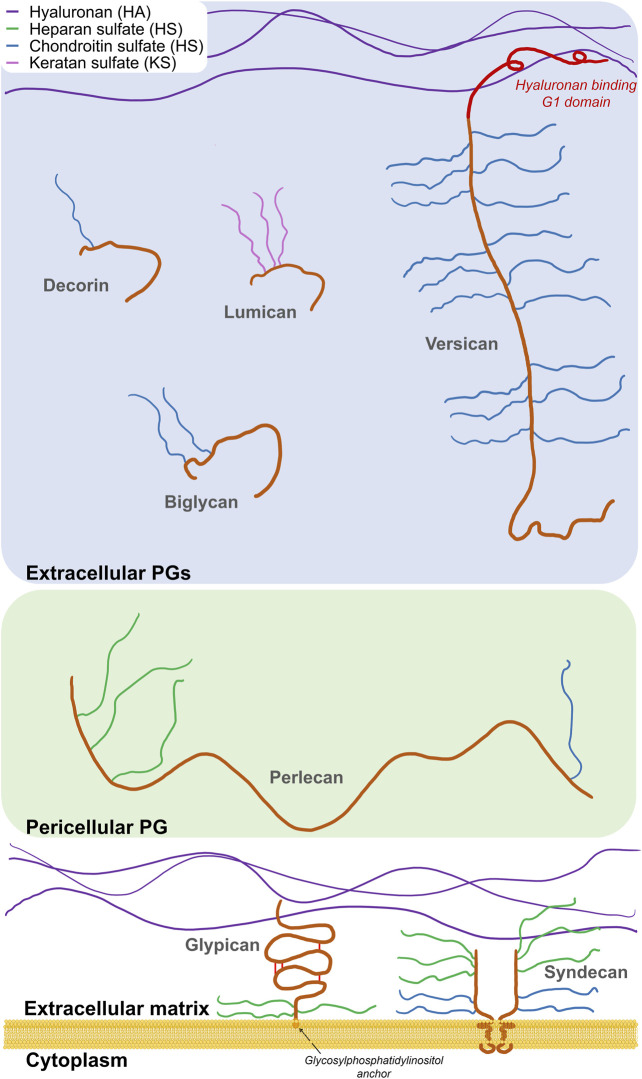
Schematic representation of the main vascular PGs according to their localization. Three main classes are reported: cell-associated, pericellular, and extracellular proteoglycans. Protein moieties are shown as orange backbones (versican G1 domain is highlighted in red).

Besides HA, that is neither sulphated nor covalently linked to a protein core, GAGs together with their protein cores form distinct PG families. PGs can be found in the ECM, in the basement membrane (pericellular PGs), associated with the cell surface (transmembrane, GPI-anchored), or inside the cells (serglycin, the only currently known) (Iozzo and Schaefer, 2015). To date, more than 20 different PGs isoforms have been identified in normal and diseased blood vessels (Wight, 2018). In vascular tissue, versican is the main CS-PG (Yao et al., 1994), whereas DS chains are found linked to decorin and biglycan, two homologous small leucine rich repeat PGs (SLRPs) (Hultgardh-Nilsson et al., 2015). KS is present in fibromodulin and lumican, two more SLRPs (Hultgardh-Nilsson et al., 2015), whereas HS is the main GAG in perlecan (Kinsella and Wight, 2005), syndecans (Gopal, 2020), and glypicans (Filmus et al., 2008). Among them, versican, decorin, biglycan, as well as fibromodulin and lumican, are classified as extracellular PGs, perlecan is a pericellular PG, whereas syndecans and glypicans are localized on the cell surface (Theocharis et al., 2016) (Figure 2).

With the exception of HA, GAGs are polymerized in the Golgi apparatus, starting from a tetrasaccharide linker consisting of xylose–galactose–galactose–uronic acid residues, by the sequential and repetitive addition of constituent monosaccharide residues. Post-synthetic chain modifications, such as epimerization of GlcA to iduronic acid and sulfation at specific positions, occur during polymerization, whereas selective removal of 6-O sulfates by specific sulfatases may occur in the extracellular space. Their structural heterogeneity, in terms of chain length, degree of iduronation, degree and pattern and sulfation, which is further increased by dynamic modifications in response to cellular and environmental stimuli, is so significant that, virtually, there are not two identical glycosaminoglycans in the body.

HA is a major constituent of the pericellular matrix and a key player in maintaining endothelial glycocalyx integrity, providing structural support and acting as signaling molecule by binding specific receptors on the cell surface. HA is synthesized by membrane-bound synthases as a high molecular weight GAG (up to 3–4 MDa), consisting of repeating residues of GlcNAc and GlcA linked each other by β-1,3- and β-1,4-glycosidic bonds. The synthesis takes place on the inner side of the plasma membrane and, during HA elongation, chains are extruded through pore-like structures to the cell surface without any further post-synthetic modifications (i.e. sulfation or epimerization) (Moretto et al., 2015). HA accumulates in aged vessels, stimulating vascular smooth muscle cells dedifferentiation and neointima formation (Moretto et al., 2015). Furthermore, during glycocalyx shedding, HA cleavage by hyaluronidase one into pro-inflammatory low molecular weight (<500 kDa) fragments contributes to endothelial derangement (Girish and Kemparaju, 2007; Vigetti et al., 2014).

## Nanofibrous Scaffold Design for Vascular Tissue Engineering

**FIGURE 3:**
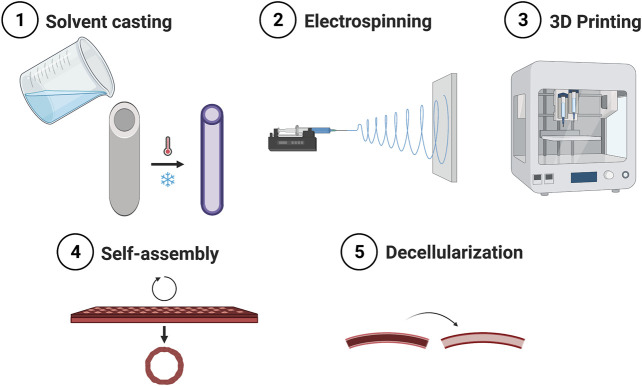
Main methods for the fabrication of small caliber vascular grafts.

A broad range of fabrication technologies are in use for the production of small diameter vascular grafts. In this section, the most used ones will be described, along with the materials used for their manufacture (Figure 3).

### Solvent Casting

Solvent casting is one of the earliest and most widely used techniques. A liquid solution of the polymer of choice for the fabrication of the final vascular graft is poured into a cylindrical mold containing an inner rod. The solution is then solidified and the system is disassembled. If polymers are dissolved in volatile solvents, then the solution is cured and the solvent is left to evaporate. Thermally induced phase separation (TIPS) can be used to introduce porosity to the scaffold, in order to improve cell infiltration. Another strategy to further modulate scaffolds’ porosity is to freeze-dry the polymer solution, by which ice crystals are formed and then sublimated leaving a pore behind. The rate of temperature decrease can influence pore size and pore connectivity. By this approach, scaffolds of silk fibroin loaded with extracellular vesicles isolated from Adipose-derived Mesenchymal Stromal Cells (ADMSC) were fabricated (Cunnane et al., 2020). These grafts presented 100% patency at the early stages of the study (4 weeks), compared to the bare scaffold and scaffold seeded with ADMCS prior to implantation. To improve mechanical strength, the lyophilized silk fibroin scaffolds were reinforced with a layer of electrospun polycaprolactone (PCL) (Gupta et al., 2020).

**FIGURE 4:**
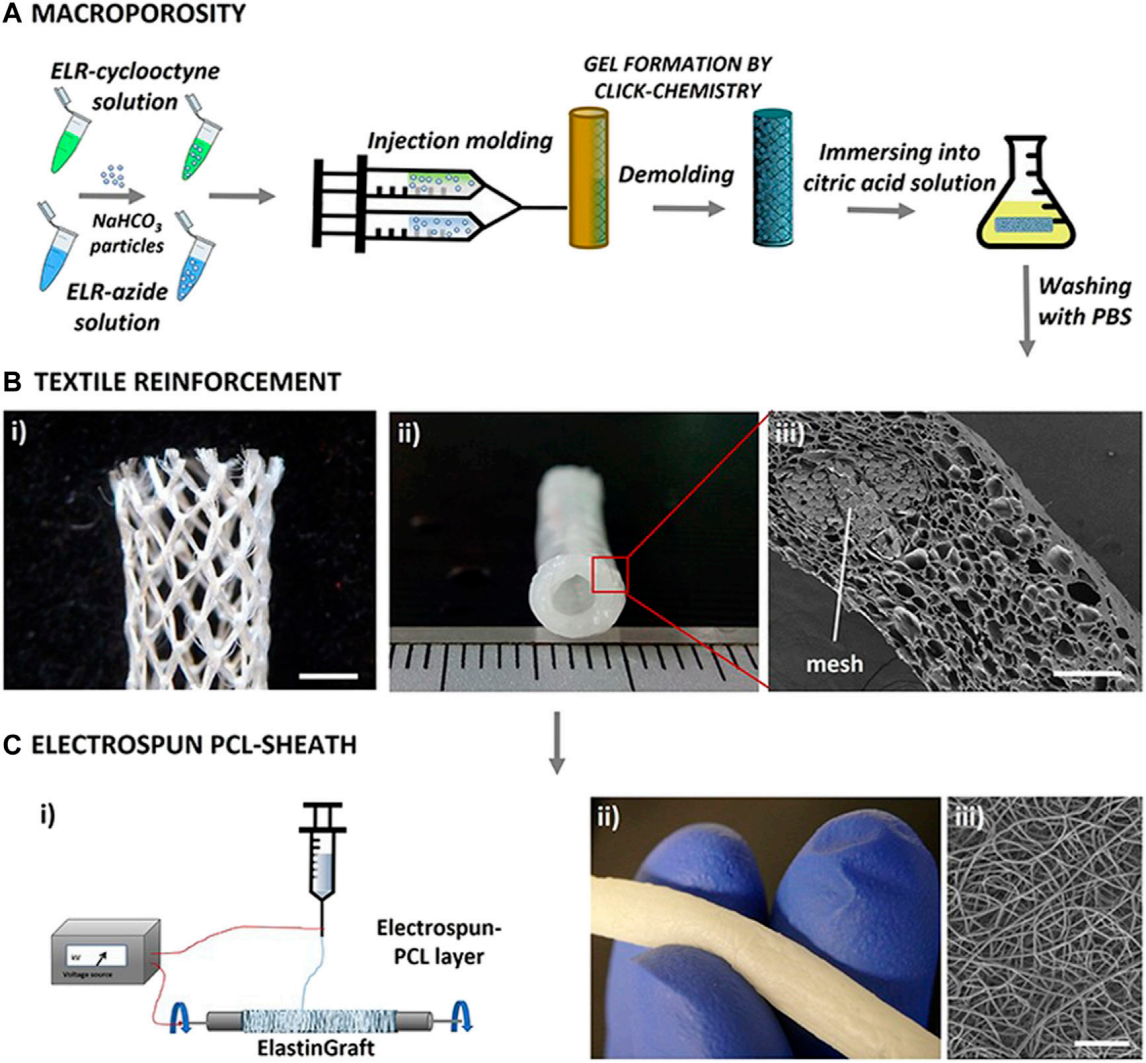
Fabrication method of porous tubular scaffolds from elastin-like recombinamers by salt leaching/gas foaming technique and electrospinning [modified from (Fernández-Colino et al., 2019)].

Porous structures can be formed also by using so-called porogens, by the salt-leaching method. As an example, salt is introduced into the polymer solution, which is then cured and immersed in water to promote the dissolution of the salt, resulting in pore formation (Figure 4). This technique was used to fabricate, for example, elastin-like recombinamer (Fernández-Colino et al., 2019) and fast degrading poly(glycerol sebacate) (PGS) (Wu et al., 2012) scaffolds with promising results. Furthermore, the modification of these scaffolds with PGS derivatives with slower degradation kinetics were shown to improve outcomes *in vivo* (Fu et al., 2020).

Another strategy to produce porous scaffolds is to use inverted colloid crystals. Microparticles of a material that is immiscible in the polymer of choice are fabricated, tightly packed in the molds, and then fused by annealing, before adding and curing the main polymer. Finally, the microparticles are dissolved, leaving a scaffold with highly interconnected pore network (Joao et al., 2014). While these techniques offer scalability and fast production, they fail to mimic the complexity of the ECM and the microenvironment in which cells are embedded.

### Electrospinning

**FIGURE 5:**
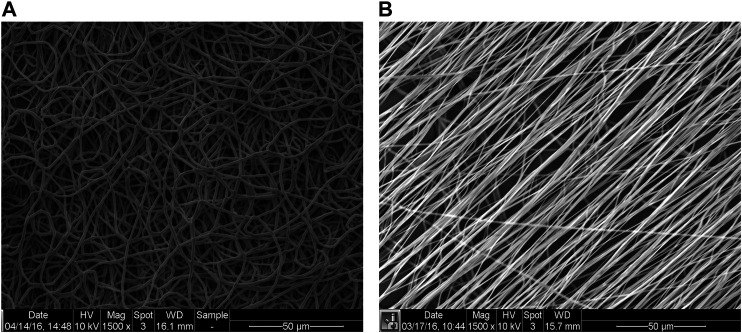
Representative scanning electron microscopy (SEM) images of random (mandrel speed = 500 rpm) and aligned (mandrel speed = 4000 rpm) PCL fibers (panels A and B, respectively) [modified from (Idini et al., 2019)].

Electrospinning has emerged as one of the most prolific techniques to fabricate vascular grafts, because of its ability to form fibrous scaffolds that replicate the approximate diameter of ECM fibers and scalability. Fibers form on a grounded collecting mandrel, or static flat stage, when a polymer solution is ejected through a charged needle. The fibers obtained with this technique can range from the nano-to the microscale, depending on polymer concentration, flow rate, applied voltage, and air gap between the spinneret and the fabrication target. The rotation speed of the collecting mandrel can be tuned to obtain fibers oriented parallel to each other or circumferentially oriented, in a similar way to the ECM in the media layer (Uttayarat et al., 2010). At low (or no) rotational speeds, the fibers are deposited in a random manner (Figure 5).

Several polymers, either of natural (e.g. fibrinogen, collagens, silk fibroin, chitosan, alginate) or synthetic [e.g. polyurethane, PCL, polylactic acid, poly(propylene carbonate)] origin, have been investigated to produce matrices composed of nanofibers for biomedical applications (Agarwal et al., 2008). Among the former, silk fibroin represents a promising substrate for vascular tissue engineering applications, due to its mechanical properties, biocompatibility and slow degradation (Marelli et al., 2010; Catto et al., 2015; Marcolin et al., 2017).

Multimaterial scaffolds can be fabricated by mixing various polymers into one syringe, by simultaneously applying voltage to different needles or by sequentially spinning different materials. The porosity of scaffolds can be improved by co-electrospinning a polymer, such as PCL, gelatin or PGS, with polyvinyl alcohol, which can be dissolved and washed away after scaffold fabrication (Khosravi et al., 2016; Tan et al., 2016). More complex scaffolds can be obtained by sequential spinning of the same material with different molecular weight (de Valence et al., 2012) or by sequentially spinning different materials, such as PCL, elastin and collagen, to obtain a tri-layered graft (McClure et al., 2010). Often, more bioactive materials have weaker mechanical properties and, thus, synthetic materials have to be added to improve their mechanics. One way to implement this is by co-axial electrospinning in a core-shell manner, in which a strong polymer, such as PCL, is used in the core and a bioactive material, such as collagen, as the shell (Duan et al., 2016). Scaffolds can be mechanically reinforced also by electrospinning an outer layer of PCL, which acts as a sheath. By this approach, Elliott et al. fabricated aligned electrospun fibrin scaffolds with a coverage of PCL, to avoid suture rupture (Elliott et al., 2019).

To improve mechanical properties of electrospun scaffolds, hybrid approaches, combining electrospinning with fused deposition modeling (FDM) (Centola et al., 2010; Wu et al., 2020) or melt electrowriting (MEW) (Brown et al., 2011) were explored too. MEW combines electrospinning and FDM, by applying a voltage to a needle and grounding the collecting surface, mostly using molten polymers and avoiding the use of solvents. MEW has been used both to include reinforcements to electrospun meshes (Jungst et al., 2019) as well as to create standalone scaffolds (Saidy et al., 2020). Other techniques were combined with electrospinning for the fabrication of vascular grafts, including TIPS of thermoplastic polyurethane (Mi et al., 2016) or poly(ester-urethane)urea (Soletti et al., 2010), or braiding and TIPS of thermoplastic polyurethane and silk (Mi et al., 2015).

### 3D Printing

3D printing relies on the accurate deposition of material in the X-Y plane while also moving, gradually, in the Z direction. 3D printing allows users to input patient-specific data and can recreate the tortuosity and multiple branching of small caliber arteries, such as the coronaries. Techniques such as 2-photon (or multiphoton) polymerization or stereolithography use light to polymerize a resin material, in a bath, by using either a focused laser or a mask, and then illuminating the whole layer. Examples of materials used are methacrylated gelatin (GelMA), methacrylated HA (Thomas et al., 2020), polyethylene glycol diacrylate, polypropylene fumarate (Melchiorri et al., 2016), polyester urethane and poly (caprolactone-co-trimethylenecarbonate) diacrylate resins (Baudis et al., 2011; Kuhnt et al., 2019; Baker et al., 2020), methacrylated poly(ethylene glycol-co-depsipeptide) (Elomaa et al., 2015).

**FIGURE 6:**
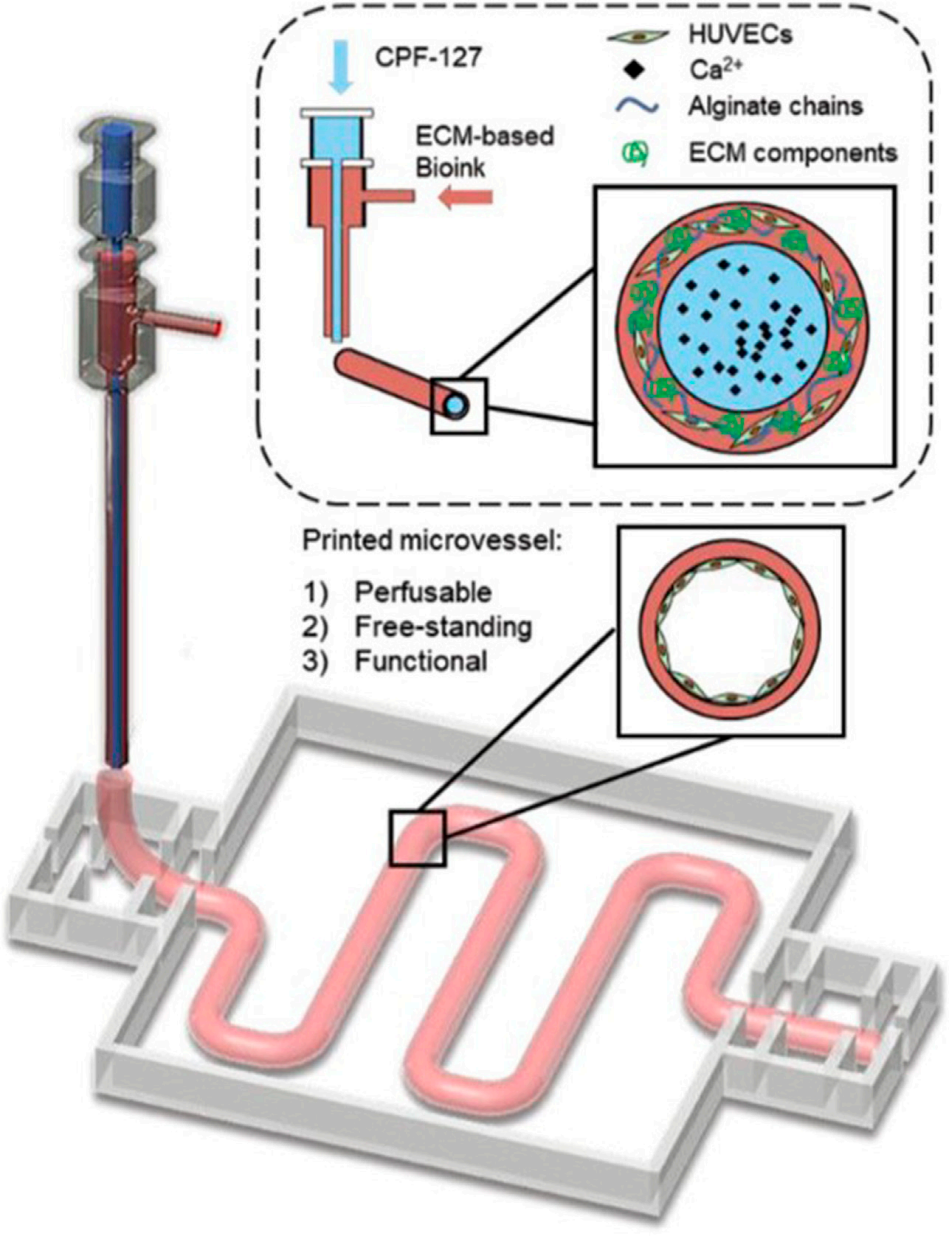
3D bioprinting of vasculature using core-shell approach, where endothelial cells are suspended in an alginate-ECM bioink as the shell and the core is a fugitive bioink composed of Pluronic F127 containing Ca^2+^ ions (CPF127) [modified from (Gao et al., 2018)].

3D bioprinting allows the spatial distribution of different materials and cell populations. By co-axially bioprinting endothelial cells (ECs) in a fugitive ink (such as gelatin or Pluronics) in the inner core and GelMA or decellularized ECM hydrogel with smooth muscle cells (SMCs) on the outer shell and then liquefying the fugitive ink upon temperature switch, a hollow endothelialized structure was obtained (Cui et al., 2019; Gao et al., 2019). This approach was developed after initial work based on co-axial printing of endothelial cells in an alginate/ECM bioink in the shell and a core consisting of Ca-containing Pluoronics fugitive ink (Gao et al., 2018) (Figure 6).

When using soft materials, such as hydrogels, traditional extrusion bioprinting might be unsuitable to create large constructs before they collapse. Strategies have been developed to overcome this limitation, by bioprinting inside a supporting bath consisting of the cross-linker and particles or gels that exhibit shear-thinning properties, which is discarded after printing (Hinton et al., 2015; Senior et al., 2019). These technique modifications have enabled the printing of large-scale cardiovascular constructs at high-resolution (Mirdamadi et al., 2020).

### Cellular Self-Assembly

Trying to overcome issues related to both scaffolds’ biodegradation by the cells and undesired immune responses, scaffold-free strategies have been developed. These vascular grafts are produced by taking advantage of the ability of cells to self-assemble and secrete ECM.

**FIGURE 7:**
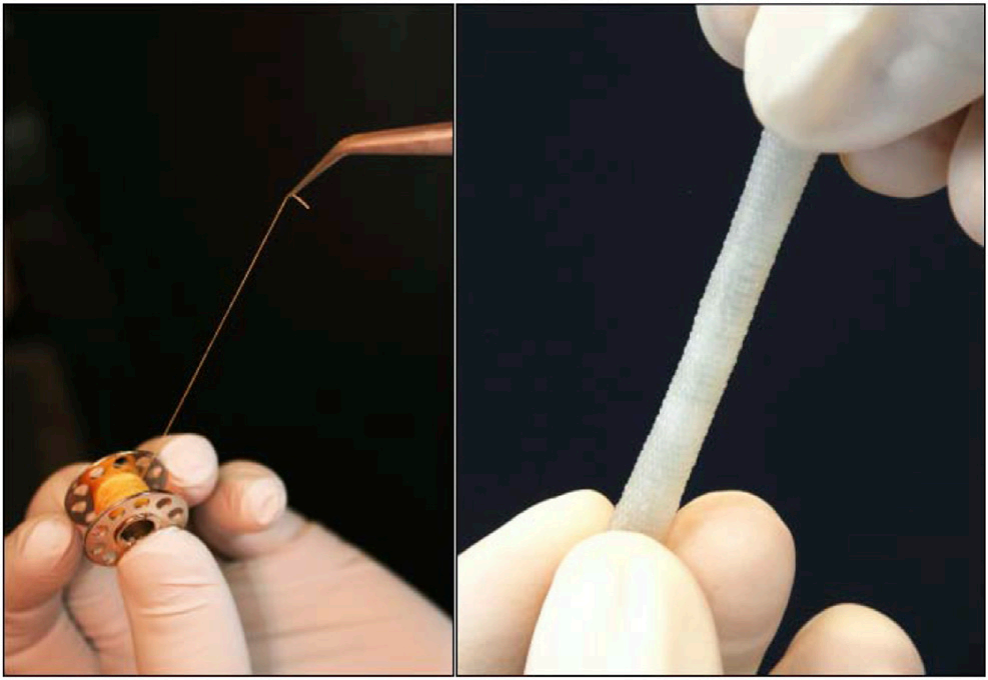
A thread (left) and a woven vessel (right) made from human Cell-assembled Extracellular Matrix [modified from (L’Heureux, 2020)].

Early work by L’Heureux demonstrated that long-term culture of fibroblasts produces an ECM that could be rolled into a tube able to withstand the physiological pulsatile flow (L'Heureux et al., 1998). Further studies showed this approach effective for arterial bypass grafting in long-term animal models (L'Heureux et al., 2006) as well as for arteriovenous shunt in hemodialysis patients (L'Heureux et al., 2007). However, a major downside of this strategy is the long culture period. The research team has recently modified its protocol by making threads from the ECM laid by the fibroblasts, which were knitted into tubes (L’Heureux, 2020; Magnan et al., 2020) (Figure 7). This method allows to produce a fully human tissue graft, with no foreign material.

Another approach to obtain implantable grafts was to seed SMCs onto fast degrading polyglycolic acid meshes and then to mature these constructs in flow bioreactors (Niklason et al., 1999; Niklason et al., 2001). To speed the process and avoid the need of harvesting autologous cells, the strategy was shifted to decellularizing the constructs once mature (Dahl et al., 2003). Such grafts were implanted in end-stage dialysis patients to treat arteriovenous fistulas (Lawson et al., 2016; Kirkton et al., 2019), as well as in patients with peripheral arterial disease (Gutowski et al., 2020). Exploiting the self-assembly approach, Itoh et al. produced hundreds of cell spheroids from a cell suspension of ECs, SMCs, and fibroblasts, and then placed them onto a needle array using a 3D printer printhead. Once the spheroids had fused into a continuous tissue, the needle array was removed and the construct placed in a perfusion reactor for maturation (Itoh et al., 2015). All these techniques require long culture times in highly controlled facilities, which makes these grafts expensive.

### Decellularized Matrices

**FIGURE 8:**
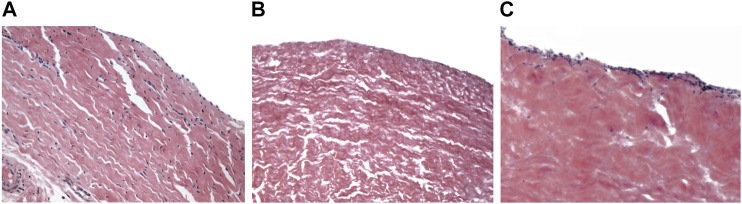
Hematoxylin and eosin staining of native bovine pericardium **(A)** and decellularized bovine pericardium before **(B)** and after **(C)** 7 days of culture with bovine fibroblasts. Magnification ×20 [from (Cigliano et al., 2012)].

Decellularized vascular grafts theoretically mimic the content and structure of native arteries more accurately than synthetic constructs. Decellularization consists in removing all cellular content while trying to minimally disrupt the ECM, using physical, chemical and/or biological methods and agents. Arteries from cadaveric donors can be employed (Madden et al., 2004; Olausson et al., 2012). Alternatively, there is the possibility of using xenogenic blood vessels, which should have fewer “availability” problems. Decellularized porcine (Tillman et al., 2012), bovine (Daugs et al., 2017) and ovine (Mancuso et al., 2014) arteries have been used as vascular grafts (Figure 8).

Decellularized matrices can also be functionalized with bioactive molecules, thus facilitating the repopulation processes as well as reducing adverse events, such as restenosis and thrombosis. In this respect, interesting results were recently obtained by Andreadis’ team that implanted a cell-free small-diameter arterial graft based on small intestinal submucosa (SIS). After coating with Hep-bound vascular endothelial growth factor, implantation in the mouse abdominal aorta and in the sheep carotid artery, showed successful endothelialization. Notably, once integrated, both mechanical properties and vascular contractility of this graft were comparable to native arteries (Koobatian et al., 2016; Smith et al., 2019; Smith et al., 2020).

Although issues were raised on the risk of inflammatory/immune reactions elicited by cell debris, including DNA fragments (Zheng et al., 2005) and alpha-Gal epitopes (Naso et al., 2012), in the last years, decellularized matrices have been widely used in different fields of tissue regeneration. Currently, there is no consensus on the criteria to be used for the decellularization methods (Ji et al., 2019).

## Application of Glycosaminoglycans to Vascular Tissue Engineering

Substitution of small-diameter vessels suffers of major problems regarding low-patency rate due to over-proliferation of smooth muscle cells (intimal hyperplasia) and thrombosis due to a lack of a functional endothelium lining the lumen of the graft. Hence, the need to design “smart substitutes” containing the chemical cues able to induce the population of the scaffold by host cells and the formation of a physiological artery wall.

Because of their pleiotropic functions and their physicochemical properties, GAGs have been extensively used in many tissue engineering applications including wound healing, as well as bone, cartilage, muscle, liver and nerve regeneration, as very recently reviewed by Sodhi H and Panitch A (Sodhi and Panitch, 2020). Furthermore, in the last 20 years, many studies have addressed the issues on small-diameter vascular regeneration by combining the most recent fabrication methods with the biofunctionalization with GAGs for the development of effective vascular grafts. We have tried to sum up the main findings of these substantial studies in two tables, according to the typology of GAG used, i.e. high and low molecular weight HA and HA derivatives (HYAFF-11) (Table 1), and Hep (Table 2). Also CS have been employed in a few studies for scaffolds functionalization (see below).

**TABLE 1:** Hyaluronan-based vascular constructs for small-caliber artery grafting.

References	Scaffold material and manufacturing method	Development level	Main findings
Turner et al. (2004)	HYAFF-11 non-woven scaffolds: Unpressed and pressed felts	*In vitro* (human saphenous vein endothelial cells)	Pressed felts: Complete endothelialization after 20 days; deposition of a subendothelial matrix containing laminin, fibronectin, type IV and type VIII collagen
Remuzzi et al. (2004)	HYAFF-11 non-woven meshes as sheets or 3D tubular scaffolds obtained by wrapping the former around a cylindrical mandrel after 7 days of culture	*In vitro* (human cell line and primary pig aortic smooth muscle cells)	Tubular scaffolds highly cellularized within the wall thickness but with lower mechanical resistance than porcine coronary arteries
Lepidi et al. (2006a)	HYAFF-11 tubes obtained by coagulation of a HYAFF-11/dmso solution around a cylindrical bar (2 mm diameter) in an ethanol bath	*In vivo* (implantation in the abdominal aorta of 15 rats)	Complete regeneration of a newly formed vascular tube at day 90; no signs of inflammation, stenoses or aneurysms; all animals survived during the 90 days follow up
Lepidi et al. (2006b)	HYAFF-11 tubes obtained by coagulation of a HYAFF-11/dmso solution around a cylindrical bar (2 mm diameter) in an ethanol bath	*In vivo* (implantation in the abdominal aorta of 30 rats)	Complete endothelialization of the tube’s luminal surface; complete vascular wall regeneration 15 days after surgery; complete degradation of the construct 4 months after implantation
Arrigoni et al. (2006)	HYAFF-11 non-woven meshes as sheets or 3D tubular scaffolds obtained by wrapping the former around a cylindrical mandrel after 7 days of culture in a medium supplemented with 50 mg/ml of sodium ascorbate	*In vitro* (primary pig aortic smooth muscle cells)	Sodium ascorbate improved cell proliferation and ECM synthesis as well as mechanical properties of the vascular construct
Zavan et al. (2008)	HYAFF-11 tubes obtained by coagulation of a HYAFF-11/dmso solution around a cylindrical bar (4 mm diameter) in an ethanol bath	*In vivo* (implantation in the carotid artery of 10 pigs)	Confirmation of the potential of hyaluronan-based graft to guide the development of a well-functioning neoartery with organized layers of elastin fibers, 5 months post-surgery; 3 cases of partial or complete occlusion by intimal hyperplasia and graft thrombosis
Pandis et al. (2010)	HYAFF-11 tubes obtained by coagulation of a HYAFF-11/dmso solution around a cylindrical bar (2 mm diameter) in an ethanol bath	*In vivo* (implantation in the vena cava of 15 rats)	Complete vein wall regeneration at day 30; complete reabsorption of the graft 4 months after surgery
Pandis et al. (2011)	HYAFF-11 patches (0.1 mm thickness)	*In vivo* (implantation in the abdominal aorta of 20 rats, rectangular breach of 1 mm × 5 mm)	Almost complete degradation of the scaffold and replacement by a neoartery wall composed of endothelial cells, smooth muscle cells, collagen, and elastin fibers organized in layers, after 16 weeks
Du et al. (2011)	Electropun aligned nanofibrous PCL scaffolds functionalized with LMW-HA by using EDC/NHS following aminolysis with 1,6-hexanediamine	*In vitro* (HUVECs)	The combination of aligned PCL fibers and LMW-HA promotes and guides the formation of a polarized functional endothelium
Zhu et al. (2014)	Human-like collagen/hyaluronic acid (HA MW 100,000–110,000 Da) composite disks obtained, at different HLC/HA ratios, by cross-linking with glutaraldehyde followed by freeze-drying	*In vitro* (human endothelial cells) and *in vivo* (subcutaneous implantation in 12 mice)	Among the different composites assessed, the 10/1 HLC/HA composite showed higher porosity, better mechanical properties and excellent biocompatibility
Yuan et al. (2016)	Highly aligned PLLA/HA (HA MW > 400 KDa) core-shell nanofibers (jet coaxial-electrospinning) crosslinked with glutaraldehyde and hydrochloric acid	*In vitro* (human umbilical arterial smooth muscle cells) and *in vivo* (implantation in the carotid artery of 6 rabbits)	Synergistic effect of nanotopographical and biochemical cues in promoting scaffold population by vSMCs and synthesis of elastin. Circumferentially aligned HA/PLLA nanofibers were effective in maintaining patency and promoting vascular regeneration during 6 weeks after surgery
Kang et al. (2019)	Electrospun scaffolds of type I collagen glycosilated with HA oligomers by reductive amination (using sodium cyanoborohydride), crosslinked with glutaraldehyde	*In vitro* (porcine iliac artery endothelial cells)	Endothelial cells proliferation was promoted by HA oligomers and inhibited by high molecular weight HA. The scaffolds had no detectable degree of hemolysis and coagulation
Jia et al. (2019)	Electrospun scaffolds of type I collagen glycosilated with HA oligomers using EDC/NHS, crosslinked with glutaraldehyde	*In vitro* (porcine iliac artery endothelial cells)	Potential of the collagen–HA electrospun nanofibers as the vascular inner-layer scaffold

HYAFF-11: 100% benzyl ester hyaluronan-based biomaterial produced by Fidia Advanced Biopolymers (Abano Terme, Italy).

**TABLE 2:** Heparin-functionalized vascular scaffolds for small-caliber artery grafting. Acellular tissue engineered vessels (A-TEVs) (highlighted in gray).

References	Scaffold material and manufacturing method	Development level	Main findings
Conklin et al. (2002)	Decellularized porcine carotid artery covalently linked with heparin using EDC	*In vivo* (implantation in the carotid artery of 2 dogs)	Excellent mechanical properties, antithrombogenicity, and tissue compatibility; effective scaffold population by both smooth muscle cells and endothelial cells within 2 months post-implantation
Tiwari et al. (2002)	Poly(carbonate-urea)urethane graft (MyoLink^™^) functionalized with arginine-glycine-aspartate (RGD) or/and hep	*In vitro* (HUVECs under pulsatile flow for 6 h)	RGD/Hep functionalization improved cell retention and metabolic activity with respect to native MyoLink
Tamura et al. (2003)	Heparinized decellularized porcine carotid artery	*In vivo* (implantation in the abdominal aorta of a dog model)	Sufficient mechanical properties and successful replacement by the host cells in 18 weeks
Conklin et al. (2004)	Decellularized porcine carotid artery, covalently linked with hep using EDC, incubated with basic fibroblast growth factor	*In vitro* (human microvascular endothelial cells or canine peripheral blood endothelial progenitor cells cultured in static and dynamic conditions)	bFGF coating on the Hep-bound decellularized grafts significantly increases attachment and proliferation of the seeded cells that remain stable under perfusion conditions
Zhou et al. (2009)	Decellularized canine carotid artery coated with hep (EDC/NHS) and coated with vascular endothelial growth factor	*In vitro* (HUVECs) and *in vivo* (implantation in the carotid artery of 15 dogs)	Complete endothelium regeneration and higher patency rate than the nonmodified scaffold after 6 months implantation
Centola et al. (2010)	Electrospun PLLA/Hep scaffolds with an outer layer of PCL by FDM	*In vitro* (hMSCs)	Drug delivery system with a microenvironment able to induce endothelial differentiation
Saitow et al. (2011)	Heparinized silk-based construct	*In vitro* (human aortic smooth muscle cells)	Stimulation of elastogenesis
Ye et al. (2011)	FGF2-loaded electrospun Hep–PCL vascular scaffolds	*In vitro* (endothelial cells)	*In vitro* (endothelial cells)
Yu et al. (2012)	Electrospun microfibres scaffolds of PLLA-PCL blends functionalized with Hep (EDC/NHS) and heparin-bound stromal cell-derived factor-1α (SDF-1α)	*In vivo* (implantation in the carotid artery of rats) and *in vitro* (culturing of explants)	Effective recruitment of endothelial progenitor cells (EPCs) to the luminal surface of the grafts, which differentiated into endothelial cells, and of smooth muscle progenitor cells, which differentiated into smooth muscle cells
Wu et al. (2012)	Poly (glycerol sebacate) core surrounded by an electrospun PCL sheath, coated with heparin	*In vivo* (implantation in the abdominal aorta of 27 rats)	Three months after implantation, the neoarteries resembled native arteries in the following aspects: Regular, strong and synchronous pulsation; a confluent endothelium and contractile smooth muscle layers; expression of elastin, collagen and glycosaminoglycan; and tough and compliant mechanical properties
Ye et al. (2012)	Electrospun Hep–PCL nonwoven tubular scaffolds	*In vitro* (human endothelial cells) and *in vivo* (implantation in the femoral artery of 2 dogs)	Low protein absorption and good cell biocompatibility; presence of endothelial cells monolayer and extracellular matrix 1 month after surgery
Zhou et al. (2012)	Decellularized canine carotid artery coated with Hep (EDC/NHS) cultured with canine endothelial progenitor cells in a custom-made bioreactor	*In vivo* (implantation in the carotid artery of 20 cell-donor dogs)	Excellent biocompatibility and high patency rate at 3 months post-implantation
Lu et al. (2013)	Polyurethane-collagen/Hep-conjugated polycaprolactone double-layer small-diameter vascular graft	*In vitro* and *in vivo* (implantation in the femoral artery of dogs)	Good biocompatibility and high patency at 8 weeks after surgery
Wang et al. (2013)	Hep-bound P(LLA-CL)/P(LLA-CL) double-layer small-diameter vascular graft	*In vitro* (endothelial cells from canine femoral vein) and *in vivo* (implantation in the femoral artery of 20 cell-donor dogs)	Biomechanical properties similar to those of canine femoral arteries; satisfactory endothelialization *in vitro*
Huang et al. (2013)	Hep-bound P(LLA-CL)/P(LLA-CL) double-layer small-diameter vascular graft pre-endothelialized	*In vitro* (endothelial cells from canine femoral vein) and *in vivo* (implantation in the femoral artery of 8 cell-donor dogs)	The pre-endothelialization has better mechanical properties and cellular compatibility than the simple heparinization
Hoshi et al. (2013)	Heparinized POC-modified ePTFE grafts	*In vitro* (platelets, primary endothelial cells, blood outgrowth endothelial cells, and smooth muscle cells)	Reduced platelet adhesion and inhibition of blood clotting; support for endothelial cells adhesion, viability, proliferation, NO production, and expression of specific markers. Smooth muscle cells increased expression of *α*-actin and decreased proliferation
Pitarresi et al. (2014)	Electrospun PHEA-eda-g-pla/pcl scaffold functionalized with Hep (EDC/NHS)	*In vitro* (human vascular endothelial cells, ECV 304)	Effective retention of bFGF and promotion of ecs growth
Yao et al. (2014)	Co-electrospun PCL/Chitosan hybrid grafts functionalized with Hep (ionic bonding with chitosan)	*In vitro* (HUVECs and human SMCs) and *in vivo* (implantation in the abdominal aorta of 9 rats)	Promotion of HUVECs growth and moderate inhibition of hSMCs proliferation; optimal anti-thrombogenic effects and enhanced *in situ* endothelialization at 1 month after surgery
Wang et al. (2015)	Small-diameter tubular PLLA/PLCL scaffolds obtained by thermally induced phase separation functionalized with Hep (EDC/NHS)	*In vitro* (pig iliac endothelial cells) and *in vivo* (subcutaneous implantation in 4 rabbits)	60% PLCL promising scaffold for engineering small-diameter blood vessel in terms of biomechanical properties; heparinization provided higher hydrophilicity, lower protein adsorption, and better *in vitro* anticoagulation properties; good cellular attachment, spreading, proliferation, and phenotypic maintenance
Jiang et al. (2015)	Decellularized rat aortas infused with poly(1,8 octanediol citrate) (POC) and functionalized with Hep (EDC/NHS)	*In vitro* (platelets, HUVECs and human SMCs)	Reduced platelet adhesion and inhibited whole blood clotting; support for endothelial cell adhesion
Dimitrievska et al. (2015)	Click-coated, heparinized, decellularized pig aortic graft	*In vitro* (platelets, HUVECs)	Reduced platelet adhesion and thrombogenicity; supported endothelial cell adhesion and proliferation
Chen et al. (2015)	Electrospun poly(l-lactic acid-co-ε-caprolactone) (P(LLA-CL)) core–shell nanofibers loaded with hep and vascular endothelial growth factor (VEGF)	*In vitro* (platelets, endothelial progenitor cells)	Effective antithrombotic potential and promotion of endothelial progenitor cells growth
Koobatian et al. (2016)	Acellular tissue engineered vessel based on small intestinal submucosa functionalized sequentially with Hep (EDC/NHS) and VEGF	*In vivo* (implantation into the carotid artery of an ovine model)	Complete endothelialization and formation of a medial layer of circumferentially aligned smooth muscle cells; high elastin and collagen content; impressive mechanical properties and vascular contractility comparable to native arteries
Shafiq et al. (2016)	Electrospun poly (l-lactide-co-ε-caprolactone) scaffolds conjugated with Hep and substance *p*	*In vitro* (platelets, human bone marrow-derived mesenchymal stem cells) and *in vivo* (subcutaneous scaffold implantation in 12 rats)	Effective host cell infiltration, neotissue formation, collagen and elastin deposition
Duan et al. (2016)	Coaxially electrospun PCL/collagen core–shell nanofibrous scaffolds crosslinked by genipin and functionalized with Hep	*In vitro* (mouse fibroblast L929 cells, ECs and SMCs)	Good biocompatibility; support for vascular cells attachment and growth on its surface, and for the infiltration of SMCs inside
Tan et al. (2016)	Co-electrospun PCL/gelatin/polyvinyl alcohol functionalized with Hep	*In vitro* (platelets, HUVECs) and *in vivo* (subcutaneous implantation in rats)	Good mechanical properties; antithrombogenic; enhanced growth of endothelial cells
Zhang et al. (2016)	Electrospun PCL/PCL2K-N3 functionalized with alkynyl-Hep	*In vitro* (rat VSMCs)	Reduced platelet adhesion; inhibition of VSMCs proliferation in a dose-dependent manner and promotion of the transition from synthetic phenotype to contractile one; moderate Hep density induces the formation of a confluent layer of contractile smooth muscle cells
Gong et al. (2016)	Decellularized rat aortas coated with electrospun PCL, with a heparinized luminal surface	*In vivo* (implantation in the abdominal aorta of 12 rats)	Satisfactory patency for up to 6 weeks; successful prevention of the occurrence of vasodilation and aneurysm formation after transplantation and reduced inflammatory cells infiltration
Fang et al. (2016)	Electropun fibrous scaffolds of elastic poly (ester urethane)urea with disulfide and amino groups (PUSN) orthogonally functionalized with Hep (EDC/NHS) and endothelial progenitor cells (EPC) recruiting peptide (TPS)	*In vitro* (platelets, mouse bone marrow-derived EPCs)	Reduced platelet deposition and improved EPCs proliferation
Jiang et al. (2016)	Decellularized rat aorta functionalized with CBP-Hep (CBP, collagen binding peptide)	*In vitro* (platelets, HUVECs)	Reduced platelet binding and whole blood clotting; stabilization of long-term endothelial cell attachment to the lumen of ECM-derived vascular conduits
Li et al. (2017)	Polycarbonate polyurethane scaffold treated with NH3 plasma and functionalized with Hep (EDC/NHS)	*In vitro* (mouse embryonic fibroblasts) and *in vivo* (implantation in the carotid artery of rabbits)	Improved *in vitro* anticoagulation and excellent biocompatibility
Zamani et al. (2017)	Composite silk-based vascular scaffold functionalized with Hep using hydroxy-iron complexes (HICs) as linkers	*In vitro* (HUVECs) and *in vivo* (subcutaneous implantation in 12 rats)	Good biomechanical properties (flexibility, suture retention strength, burst pressure, and compliance); sustained antithrombogenicity, cytocompatibility and nonhemolytic properties
Hsieh et al. (2017)	Electrospun poly-l-lactide-co-caprolactone (PLCL) microfiber vascular grafts aminolyzed with plasma treatment or fmoc-peg-diamine insertion for Hep conjugation	*In vivo* (subcutaneous implantation in rats)	Plasma treatment resulted in significantly higher initial hep density and higher Hep stability on PLCL microfibers than fmoc-peg-diamine treatment as well as better mechanical properties; Hep coating with both methods promoted cell infiltration
Braghirolli et al. (2017)	Electrospun polycaprolactone scaffolds functionalized with Hep (EDC/NHS) and absorbed VEGF	*In vitro* [human EPCs or mesenchymal stem cells (MSCs)]	Mechanical properties compatible with the native arteries; antithrombogenic properties and increased EPC proliferation, favoring the formation of the endothelial layer
Qiu et al. (2017)	Electrospun polycarbonate-urethane (PCU) nanofibrous grafts treated with plasma to conjugate Hep *via* end-point immobilization	*In vivo* (implantation in a rat common carotid artery anastomosis model)	High patency rate at 2 and 4 weeks; complete endothelialization of the luminal surface with an aligned, well-organized monolayer of endothelial cells, extensive graft integration in terms of vascularization and cell infiltration from the surrounding tissue
Henry et al. (2017)	Electrospun PLLA scaffold blended with low MW PCL or low MW PLLA functionalized with Hep (EDC/NHS) and absorbed VEGF	*In vivo* (implantation in a rat common carotid artery model)	Enhanced endothelium formation and the overall patency of vascular grafts; increased cell infiltration into the electrospun grafts and production of collagen and elastin fibers within the graft wall
Lee et al. (2017)	PCL functionalized with Hep–tyramine polymer and a potent anti-neointimal drug (mitogen activated protein kinase II inhibitory peptide; MK2i)	*In vitro* (platelets, VSMCs)	Enhanced blood compatibility with significantly reduced protein absorption and platelet adhesion; significant inhibitory effects on VSMC migration associated with intimal hyperplasia
Hu et al. (2017)	Coaxial-elctrospun scaffolds of poly (l-lactide-co-caprolactone) [P(LLA-CL)]/collagen/elastin with hep and VEGF encapsulated in the core	*In vitro* (human aortic endothelial cells) and *in vivo* (implantation into a rabbit infrarenal aortic replacement model)	High attachment efficiency and proliferation; high short-term patency
Caracciolo et al. (2017)	Electrospun scaffolds from blends of poly (l-lactic acid) (PLLA) and segmented polyurethane (PHD) functionalized with lysozyme/heparin (EDC/NHS)	*In vitro* [human adipose-derived stem cells (MSC)]	Inhibition of platelet adhesion and of hemolysis; adhesion and proliferation of human adipose-derived stem cells; antimicrobial activities
Cao et al. (2017)	Electrospun PCL scaffolds aminolyzed and functionalized with hep (EDC/NHS)	*In vitro* (rabbit SMCs)	Induction of SMCs penetration into the scaffold and differentiation into contractile phenotype
Jiang et al. (2017)	Rat aorta decellularized vascular graft functionalized with antioxidant poly(1, 8-octamethylene-citrate-co-cysteine) (POCC) and Hep	*In vivo* (implantation in a rat aorta model)	Grafts displayed antioxidant activity, patency, and minimal intramural cell infiltration with varying degrees of calcification (inversely related to the antioxidant capacity), at 3 months post-surgery
Zhou et al. (2018)	Dual layer conduit consisting of collagen I-hyaluronic acid (external layer) and collagen I-Hep (inner layer) crosslinked with EDC	*In vitro* [fibroblast cell (Cos-7) and human microvascular endothelial cells (HMEC)]	Satisfactory mechanical performance and support for cells adhesion, proliferation and elongation
Wan et al. (2018)	Electrospun PCL/keratin nanofibrous mats functionalized with Hep (EDC/NHS)	*In vitro* (platelets, HUVECs, human umbilical artery smooth muscle cells (HUASMCs))	Effective antithrombotic potential; induction of NO release, which enhance endothelial cell growth and inhibits smooth muscle cell proliferation and platelet adhesion
Xu et al. (2018)	Electrospun PCL scaffolds functionalized with Hep (EDC/NHS)	*In vivo* (implantation in infrarenal abdominal aorta of 30 rats)	All implanted grafts were patent during the 6 months post-surgery and showed a well-organized neo-tissue with endothelium formation, smooth muscle regeneration, and extracellular matrix formation
Norouzi and Shamloo (2019)	Bilayer heparinized vascular graft: Inner layer made by co-electrospinning of PCL and gelatin; outer layer fabricated by freeze-drying of gelatin hydrogel; Hep blending in gelatin solution and emulsion of PCL fibers	*In vitro* (HUVECs and rat VSMCs)	Improved endothelial cell attachment and decreased amount of activated platelets; mechanical properties similar to the coronary artery
Cai et al. (2019)	Decellularized porcine carotid arteries functionalized with Hep (EDC/NHS)	*In vivo* (subcutaneous implantation in 8 rats)	Improved mechanical properties, reduced inflammatory reaction and slow degradation time; effective inhibition of thrombogenesis
Kuang et al. (2019)	Two-layer composite vascular graft: Inner layer made of poly(lactic-co-glycolic acid)/Collagen nanofibers modified by mesoporous silica nanoparticles and grafted with polyethylene glycol and Hep; outer layer made of polyurethane nanofibers	*In vitro* (HUVECs) and *in vivo* (implantation into rabbit carotid artery)	Good blood compatibility; absence of inflammatory reaction; regeneration of endothelial cells monolayer and smooth muscle media layer
Smith et al. (2019)	Acellular tissue engineered vessel based on small intestinal submucosa functionalized sequentially with Hep (EDC/NHS) and VEGF	*In vivo* (implantation in abdominal aorta of mice)	Well-demarcated luminal and medial layers resembling those of native arteries; anti-inflammatory action of VEGF on infiltrating monocytes
Wang D. et al. (2019)	PLLA/PLGA/PLCL composite scaffolds fabricated by using TIPS, functionalized with Hep (EDC/NHS) and stromal cell-derived factor-1 alpha (SDF-1α)	*In vitro* (rat EPCs, HUVECs and hVSMCs)	Enhanced anticoagulation of vascular scaffold; acceleration of endothelialization and inhibition of hVSMCs proliferation
Jin et al. (2019)	Double-layer vascular scaffold: Inner layer made of electrospun end-group heparinized PCL nano- and microfibers; outer layer made of electrospun PCL	*In vitro* (HCs and SMCs) and *in vivo* (implantation into carotid artery of 6 rabbits)	Patency, endothelialization and fine revascularization were observed at 2 months post-implantation; aneurysmal dilatation of the outer layer; no signs of calcification
Wang W. et al. (2019)	Electrospun PCL scaffolds functionalized with multiple layers of vascular endothelial growth factor (VEGF) and Hep (repeated electrostatic adsorption self-assembly) crosslinked by genipin	*In vitro* (platelets, HUVECs)	Early and full release of VEGF to promote rapid endothelialization; gradual but sustained release of Hep for long-term anticoagulation and antithrombogenicity; improved cell viability and rapid endothelialization
Shi et al. (2019)	Electrospun PCL/gelatin hybrid vascular grafts functionalized with Hep (EDC/NHS)	*In vivo* (implantation in abdominal aorta of 18 rats)	Promotion of endothelialization and regulation of smooth muscle regeneration; inhibition of thrombosis
Wang et al. (2020)	Electrospun PCL scaffolds functionalized with multiple layers of vascular endothelial growth factor (VEGF), polylysine, and Hep (repeated electrostatic adsorption self-assembly) nanoparticles crosslinked by genipin	*In vitro* (platelets, HUVECs)	Successful induction of vascular endothelialization and long-term anticoagulation; long-term release of bioactive factors without burst release
Smith et al. (2020)	Acellular tissue engineered vessel based on small intestinal submucosa functionalized sequentially with Hep (EDC/NHS) and VEGF	*In vivo* (implantation into the carotid artery of an ovine model)	Immobilized VEGF captures blood circulating monocytes that differentiate into mature ECs that align in the direction of flow and produce nitric oxide under high shear stress. Highly prevalent circulating MC contribute directly to the endothelialization of acellular vascular grafts under the right chemical and biomechanical cues

### Hyaluronan-Based Vascular Constructs

Since discovery in 1934, HA has been extensively studied, and thanks to its viscoelastic properties, physiological activity, and biocompatibility, it has been used in a wide range of medical fields from orthopedics to cosmetics (Abatangelo et al., 2020). Due to the high-water solubility and the propensity to form very viscous solutions, its use in vascular tissue engineering requires the crosslinking with other natural (e.g. type I collagen) or synthetic materials (e.g. PCL) or its chemical modification to obtain semisynthetic polymers (Table 1). Among the latter, HYAFF-11, an HA derivative obtained by esterification with benzyl alcohol, was proven to be useful for small-diameter vascular grafts fabrication, also in a large animal model. This polymer is biocompatible, bioresorbable, able to interact with polar molecules, and it can be processed to obtain several types of devices such as tubes, membranes, non-woven fabrics, gauzes, and sponges (Vindigni et al., 2009). HYAFF-11 tubes, obtained by coagulation of a HYAFF-11/DMSO solution around a cylindrical bar in an ethanol bath, were successfully implanted in the abdominal aorta of 30 rats (2 mm diameter, 1 cm length) (Lepidi et al., 2006a; Lepidi et al., 2006b), as well as in the carotid artery of 10 pigs (4 mm diameter, 5 cm length) (Zavan et al., 2008), as temporary absorbable guides to promote regeneration of vascular structures. These studies showed the potential of these HA-based grafts to guide the development of a functional neo-artery, consisting of a confluent endothelium lining the luminal surface and a vascular wall with organized layers of elastin fibers. Interesting *in vitro* results were also obtained with electrospun PCL or collagen type I scaffolds functionalized with either high or low molecular weight HA, showing excellent biocompatibility and the potential to guide the formation of a polarized functional endothelium (Table 1). Recently, Yuan et al. (Yuan et al., 2016) obtained highly aligned HA/PLLA nanofibers in core-shell structure by coaxial stable jet electrospinning, which was shown effective in inducing human umbilical arterial smooth muscle cells elongation, orientation, proliferation, and differentiation toward a contractile phenotype. Circumferentially aligned HA/PLLA nanofibers scaffolds were successfully implanted in the carotid artery of six rabbits, showing high patency and efficacious vascular regeneration, 6 weeks post-surgery (Yuan et al., 2016).

### Heparin-Based Vascular Constructs

Since its discovery in 1916, Hep has been widely clinically used to treat thrombotic disorders, as it amplifies the inhibitory activity of antithrombin III toward thrombin by facilitating the formation of a ternary complex with factor Xa. Although Hep and HS share the same biosynthetic pathway, the former has higher degree of sulfation (2.6 vs. 0.6 sulfate groups per disaccharide) and epimerization (up to 90 vs. 20% iduronation) (Liu and Linhardt, 2014). Initially known for its anticoagulant properties, many evidences showed Hep’s usefulness as anti-inflammatory agent in the treatment of venous thromboembolism and other vascular diseases. Hep’s pleiotropic roles include the inhibitory activities toward neutrophils functions, endothelial activation and smooth muscle cells proliferation (Yang et al., 2012; Poterucha et al., 2017), well-known key events in atherogenesis and vascular graft failure. Due to its capacity to bind and release growth factors, such as VEGF and bFGF, and to modulate angiogenesis, together with its anti-thrombotic properties, Hep has been used to functionalize many different systems including hydrogels, films, and electrospun fibers (Aslani et al., 2020). Table 2 summarizes the multitude of studies, published in the last 20 years, that exploited the angiogenic regulatory activities of Hep for the development of effective small diameter vascular grafts. Among them, the great majority developed electrospun scaffolds made of exclusively synthetic (e.g. poly-Ɛ-caprolactone, poly-L-lactide-co-caprolactone, poly-L-lactic acid) or hybrid (containing collagen, silk, chitosan, keratin, etc.) mats functionalized with Hep, mainly *via* chemical crosslinking using N-Ethyl-N′-(3-dimethylaminopropyl) carbodiimide/N-hydroxy succinimide (EDC/NHS). Besides, acellular tissue engineered vessels (A-TEVs) consisting of vascular tissue (mainly carotid artery or aorta segments from pig, dog, or rat) or small intestinal submucosa, undergone a decellularization process and, subsequently, coated with Hep and, in some cases, angiogenic growth factors, have been fabricated.

Most of the reported studies, performed *in vitro* by culturing either endothelial cells or smooth muscle cells, showed good mechanical properties, enhanced biocompatibility and anti-thrombogenicity of these grafts, and the potential of Hep to promote endothelialization and smooth muscle cells differentiation toward a contractile phenotype. Besides, some interesting results were obtained *in vivo* with both synthetic grafts and A-TEVs.

Wu et al. (Wu et al., 2012) designed a cell-free fast-degrading elastomeric graft consisting of a PGS core surrounded by a PCL sheath, coated with Hep, which was successfully implanted in the abdominal aorta of 21 rats (porous PCL scaffolds were implanted in six rats as controls). The graft was rapidly substituted by a neo-artery with mechanical and functional properties very similar to those of the native one.

To improve endothelialization and reduce thrombotic events and restenosis, Song’s team developed two methods for optimal surface functionalization of synthetic grafts with Hep. In the first one, electrospun polycarbonate-urethane nanofibrous scaffolds underwent plasma treatment followed by Hep conjugation *via* end-point immobilization. Grafts were implanted in a rat common carotid artery anastomosis model, showing high patency rate and extensive graft integration (Qiu et al., 2017). In the second one, electrospun scaffolds were obtained using blends of high and low molecular weight elastomeric polymers to improve both functionalization and mechanical properties of the grafts. PLLA scaffolds with 5% low molecular weight PCL were functionalized with Hep, loaded with VEGF and implanted into the carotid artery of rats, evidencing a synergistic effect of these two angiogenic factors on graft patency, endothelialization, and neo-artery formation (Henry et al., 2017).

As above mentioned, Andreadis’ team designed an A-TEV from decellularized SIS, coated it sequentially with Hep and VEGF, and then implanted it in both mouse abdominal aorta and sheep carotid artery (Koobatian et al., 2016; Smith et al., 2019; Smith et al., 2020). They obtained promising results including complete endothelialization and formation of a medial layer of circumferentially aligned smooth muscle cells, high elastin and collagen content, impressive mechanical properties and vascular contractility comparable to native arteries. They showed that VEGF was essential for modulating the inflammatory response of monocytes and the regeneration of a functional artery wall (Smith et al., 2019). Additionally, they demonstrated that VEGF was able to capture circulating monocytes that differentiated into mature endothelial cells, therefore directly contributing to the endothelialization of acellular vascular grafts (Smith et al., 2020).

### Chondroitin Sulfate-Based Vascular Constructs

Differently from both HA and Hep, only few *in vitro* studies dealt with usefulness of CS for functionalization of small-diameter vascular grafts. In particular, Lerouge’s team functionalized poly(ethylene terephthalate) scaffolds with CS (*via* EDC/NHS) and assessed their functionality culturing HUVECs and VSMCs from rat embryonic thoracic aorta. Overall, these studies showed that CS coating prevented platelet adhesion and activation, while promoting HUVECs growth and resistance to flow-induced shear stress, and survival and inward penetration of VSMCs (Thalla et al., 2014; Savoji et al., 2017). The paucity of these studies could be related with CS implications in atherogenesis. In fact, CS is a major GAG found in both normal vessels and atherosclerotic lesions that participates in apolipoprotein B100 binding, therefore contributing to lipids accumulation into the subendothelial space of arteries. These events are mediated by specific interactions between a proteoglycan-binding site in Apolipoprotein B100, consisting of a few basic amino acids, and the negatively charged CS (Boren et al., 1998), with increasing affinity for over-sulfated chains (Sambandam et al., 1991).

## Conclusion

GAGs represent key players in vascular physiology as well as in the pathogenesis of atherosclerosis. A deep knowledge of GAGs structural complexity, which accounts for their numerous functions, still represents a challenge but also a huge potential source of information to pave the way to vascular tissue engineering. In this review, we have provided readers with an overview of the numerous efforts performed in the last 20 years, in the attempt to develop functional resorbable scaffolds for small-diameter vascular regeneration, using GAGs as molecular cues to guide the correct endothelialization of the luminal surface and neo-artery formation. Although many progresses have been obtained both *in vitro* and *in vivo* using HA, HA derivatives, or Hep as bioactive molecules, none of the mentioned devices has reached the clinical trial yet, leaving the field open to further studies, including those exploring the effects of specific sulfated saccharide sequences or synthetic glycans related molecules on vascular regeneration.
